# MiR-1 suppresses tumor cell proliferation in colorectal cancer by inhibition of Smad3-mediated tumor glycolysis

**DOI:** 10.1038/cddis.2017.60

**Published:** 2017-05-04

**Authors:** Wanfu Xu, Zijing Zhang, Kejian Zou, Yang Cheng, Min Yang, Huan Chen, Hongli Wang, Junhong Zhao, Peiyu Chen, Liying He, Xinwen Chen, Lanlan Geng, Sitang Gong

**Affiliations:** 1 Department of Gastroenterology, Guangzhou Women and Children’s Medical Center, Guangzhou Medical University, Guangzhou 510623, China; 2 Guangzhou Institute of Paediatrics, Guangzhou Women and Children’s Medical Center, Guangzhou Medical University, Guangzhou, Guangdong 510623, China; 3 Wuhan Institutes of Virology, Chinese Academy of Sciences, Wuhan, Guangdong 510623, China; 4 Department of General Surgery, Hainan General Hospital, Haikou, Hainan, China

## Abstract

Aberrant expression of microRNA (miR)-1 has been observed in many human malignancies. However, the function and underlying mechanism of miR-1 remains elusive. To address the specific role of miR-1 in tumor glycolysis using the gain- or loss-of-function studies. Metabolic studies combined with gene expression analysis were performed *in vitro* and *in vivo.* We demonstrated aberrant expression of miR-1 in aerobic glycolysis, the Warburg effect, in cancer cells. MiR-1 suppressed aerobic glycolysis and tumor cell proliferation via inactivation of Smad3 and targeting HIF-1*α*, leading to reduce HK2 and MCT4 expression, which illustrated a novel pathway to mediate aerobic glycolysis in cancer cells. Overexpression of miR-1 mimics significantly decreased tumor glycolysis, including lactate production and glucose uptake, and cell proliferation, and these effects were reversed by ectopic expression of Smad3. Importantly, endogenous Smad3 regulated and interacted with HIF-1*α*, resulting in increasing activity of Smad3, and this interaction was dramatically abolished by addition of miR-1. We further demonstrated that Smad3 was central to the effects of miR-1 in colorectal cancer cells, establishing a previously unappreciated mechanism by which the miR-1/Smad3/HIF-1*α* axis facilitates the Warburg effect to promote cancer progression *in vitro* and *in vivo*. The results indicate that miR-1 may have an essential role as a tumor suppressor, suggesting its potential role in molecular therapy of patients with advanced colorectal cancer.

Aerobic glycolysis, a phenomenon described by Otto Warburg almost 90 years ago, is a hallmark of cancer. The Warburg effect refers to the heavy reliance of cancer cells on glycolytic fermentation for energy provision regardless of ambient oxygen tension.^1^ There is no doubt that in cancer cells, metabolic reprogramming promotes rapid proliferation and apoptosis resistance. The glycolytic pathway comprises a series of 10 metabolic reactions catalyzed by multiple enzymes or enzyme complexes, including hexokinases and phosphofructokinases, such as HK2 and PFKP, whose expression is often increased in tumor cells, resulting in a rapid shift from glucose uptake to lactate production.^2^ Accumulation of lactate in tumor microenvironment contributes to immune escape, migration, tumorigenesis and angiogenesis,^3^ suggesting a functional role of the Warburg effect in tumor progression. Elucidating the mechanisms underlying glycolysis in cancer cells might reveal new candidate therapies of human malignancies.

Transforming growth factor (TGF-*β*)-Smad2/3 signaling regulates a number of activities involved in colorectal cancer progression. TGF-*β* signaling is initiated by binding to its type II receptor (TGF-*β*RII), which subsequently activates the type I receptor (TGF-*β*RI) and phosphorylates Smad2/3 at a highly conserved C-terminal SSXS motif. Together with Smad4, Smad2 and Smad3 function as reprogramming factors to regulate cancer development in various ways. For example, the growth-inhibitory effect of the TGF-*β*-Smad3 pathway in initiation of tumor development and promotion of advanced tumors mediated by ZEB1, Twist, Slug and Snail-induced epithelial–mesenchymal transition, invasion, and metastasis, indicating Smad3 is critical to TGF-*β* signaling.^4, 5, 6, 7^ In addition, phosphorylation of Smad3 linker regions, including Ser213, Ser204, Ser208 and Thr179, is an early event in colitis-associated colorectal cancer, and indirectly inhibits its COOH-terminal phosphorylation and subsequently suppresses tumor-suppressive pSmad3C signaling, generating resistance to the growth-inhibitory effect of TGF-*β*.^6, 8, 9, 10, 11, 12^ A recent report by Yadav *et al.* illustrated that conditional Smad3 knockout induced white fat to brown fat phenotypic transition and promoted mitochondrial biogenesis and function in white adipose tissue by regulating the PGC-1*α* promoter and PRDM16 target genes.^13^ This suggests an important role of Smad3 in regulating glucose and energy homeostasis, which is consistent with the finding by Sun *et.al*.^14^ and Tan *et.al*.^15^ Nevertheless, it should be noted that regulation of tumor glycolysis in cancer cell metabolism has never been directly tested in colorectal cancer cells.

Evidence for the regulation of tumor cell glycolysis by microRNA (miRNAs) is increasing.^16, 17, 18, 19, 20^ miRNAs are a group of small, noncoding, endogenous RNAs that negatively regulate target genes, usually by imperfect complementation sequence pairing to the 3′-untranslated region (UTR) of the target genes. The pairing results in either mRNA degradation or translational repression and control of an aspect of tumor progression. A link between miR-1 and glycolysis was first reported by Wei *et al.* who showed that miR-1 repressed the heart fetal gene program by directly targeting estrogen-related receptor beta (Err*β*), a principal regulator of glycolysis and glycogenesis.^21^ This discovery may be relevant to understanding mechanisms of oncogenesis, as miR-1 is a well-known tumor suppressor, acting by inhibition of multiple targets, including TAGLN2, CCND2, CXCR4 and SDF-1R,^22, 23, 24, 25, 26^ which play key roles in tumor progression. Downregulation of miR-1 in tumor tissue is associated with worse clinical prognosis, but little is known about miR-1-mediated regulation of cancer metabolism.

We aimed to determine whether, and how, aberrant expression of miR-1 might reprogram cancer metabolism. We found that miR-1 suppressed cancer cell proliferation by regulation of Smad3-mediated aerobic glycolysis. We also revealed that the miR-1/Smad3 axis regulated cancer cell metabolism by targeting HIF-1*α* or by an HIF-1*α*-dependent mechanism including a positive loop by which Smad3 interacts with and regulates HIF-1*α*. Our results established a previously unappreciated mechanism by which the miR-1/Smad3-HIF-1*α* axis suppressed the Warburg effect and cancer progression.

## Results

### MiR-1 was weakly expressed in colorectal cancer

**Figure 1:**
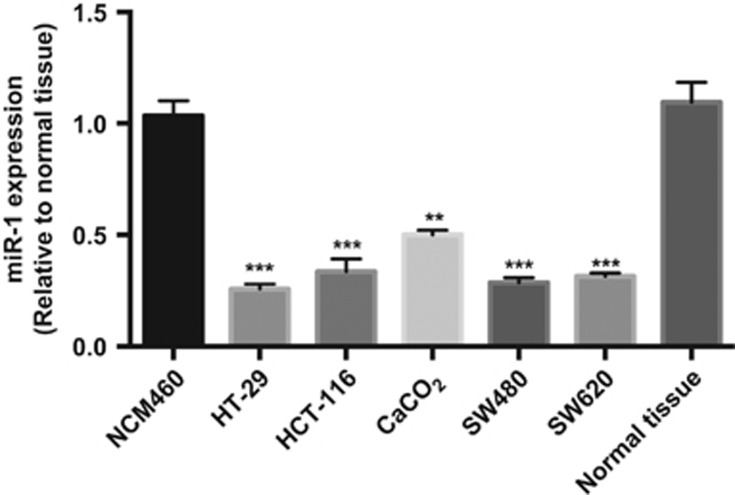
The expression of miR-1 is downregulated in CRC cell lines. (**a**) The relative expression of miR-1 in five CRC cell lines (SW480, SW620, HCT-116, CaCO_2_ and HT-29) was significantly decreased compared with NCM460 and normal tissue by real-time PCR, Data were presented as mean±s.d. **P<0.01 and ***P<0.001, *n*=4, One Sample *t*-test

The biological function of MiR-1 in colorectal cancer is not clear. We detected miR-1 expression using qRT-PCR in colorectal cancer cell lines. As shown in Figure 1a, the results revealed that miR-1 expression in the SW480, SW620, HCT-116, HT-29 and CaCO_2_ of colorectal cancer cell lines was significantly downregulated compared with the normal human colon epithelial cell line NCM460 or the normal tissue of non-cancerous tissue samples.

### MiR-1 inhibited cell proliferation *in vitro*

**Figure 2:**
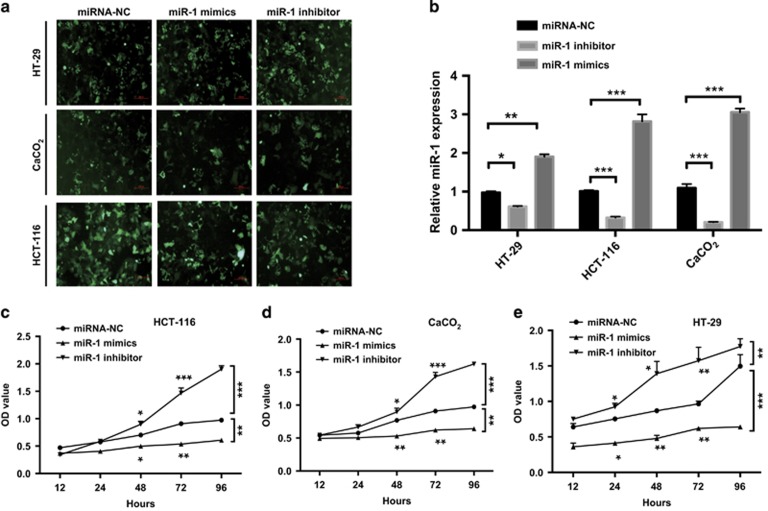
miR-1 suppresses cell proliferation. (**a**) Representative fluorescence images of colorectal cancer cells, HCT-116, HT-29 and CaCO_2_, infected lentivirus as indicated, and (**b**) RT-PCR analysis was performed to verify miR-1 expression level, Data are expressed as mean±s.d., *n*=3 in each group. *P<0.05; **P<0.01; ***P<0.001 between groups using one sample *t*-test. (**c**–**e**) the time course of miR-1 on cell proliferation was evaluated by CCK-8 assay in HCT-116, CaCO_2_ and HT-29 cells, respectively. Data were presented as mean (±s.d.). *n*=3 in each group, *P<0.05; **P<0.01; ***P<0.001 *versus* control using unpaired Student’s *t*-test

Recently, Wei *et al.* showed by massively parallel sequencing that a large proportion of genes upregulated after deletion of miR-1 s were associated with the cardiac fetal gene program and fetal sarcomeres and regulated cell proliferation, glycolysis or glycogenesis,^21^ suggesting that miR-1 has a key role in regulation of energy metabolism. To test that hypothesis and investigate functional relationships, we established HCT-116, HT-29 and CaCO_2_ cells that stably expressed miR-1 mimics or miR-1 inhibitor by virus infection and confirmed by qPCR assay (Figures 2a and b). Cell viability was significantly reduced in colorectal cancer cells expressing miR-1. The contrasting results obtained in cells expressing miR-1 inhibitor (Figures 2c–e) demonstrated that miR-1 had a consistent anti-proliferative role in HCT-116, HT-29 and CaCO_2_ of colorectal cells.

### MiR-1 suppressed glycolysis in colorectal cancer cells

The above result showed that miR-1 inhibited cell proliferation in colorectal cancer cells; it is still, however, unclear whether the action of miR-1 in tumor cell proliferation by restraint of tumor glycolysis. Measurement of tumor metabolic parameters revealed that cellular lactate production and glucose uptake were significantly decreased in culture of HCT-116, HT-29 and CaCO_2_ cells overexpressing miR-1 mimics. At the same time, glucose uptake and lactate production were increased in cells expressing miR-1 inhibitor (Figures 2f–h). The results thus indicated that miR-1 mimics could inhibit glycolysis, and taken together, the gain- or loss-of-function studies led to the conclusion that miR-1 suppressed aerobic glycolysis, or the Warburg effect, in colorectal cancer cells, which further inhibited cell proliferation.

### MiR-1 significantly decreased glycolytic relative gene expression

**Figure 3:**
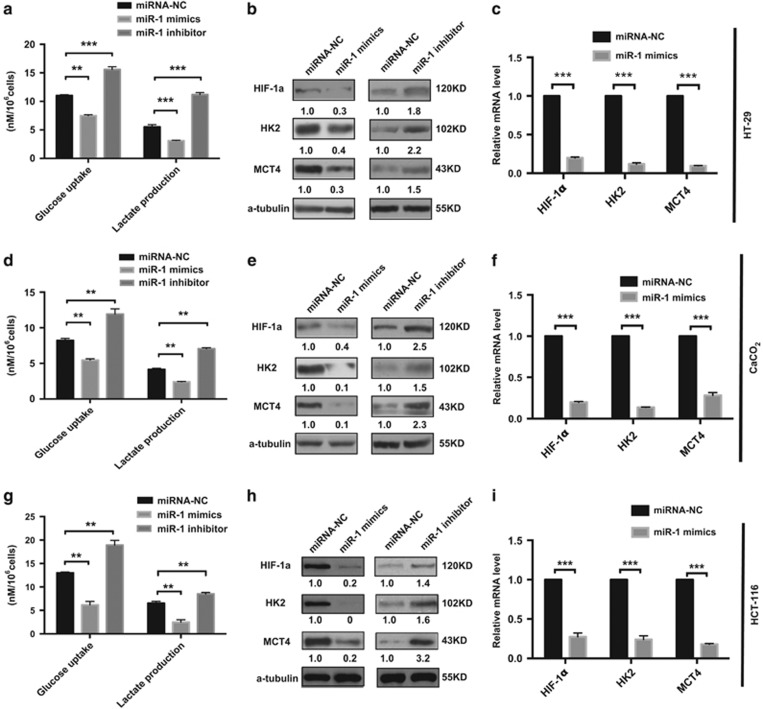
miR-1 inhibits tumor glycolysis and key enzymes expression. (**a**, **d**, **g**) the effect of miR-1 on the concentrate of glucose consumption and lactate production in colorectal cancer cells was measured as described in Material and Methods and difference between group analyzed by using unpaired Student’s *t*-test, *n*=3, **P<0.01; ***P<0.001 *versus* miRNA-NC. (**b, e, h**) Protein levels of metabolic enzymes in the Warburg effect were determined by western blot in HT-29, HCT-116 and CaCO_2_ cells overexpressing miRNA-NC, miR-1mimics and miR-1 inhibitor. *α*-tubulin served as loading control, for each marker band, values were normalized to internal control and the density of control was set at 1.0. (**c**, **f**, **i**) Indicated gene mRNA levels was measured by Real-time PCR in HT-29, HCT-116 and CaCO_2_ cells expressing NTC or miR-1 mimics. Data were presented as mean (±s.d.). *n*=3 in each group. ***P<0.001 (One sample *t*-test)

The above result we found that miR-1 negatively regulated tumor glycolysis and inhibited cell proliferation, which force us to investigate the effect of miR-1 on expression of multiple glycolytic enzymes. Cells transfected with miR-1 mimics significantly suppressed HK2, HIF-1*α* and MCT4 protein expression in HCT-116, HT-29 and CaCO_2_ cells. In contrast, the presence of miR-1 inhibitor increased glycolytic protein expression in HCT-116, HT-29 and CaCO_2_ cells (Figures 3a, c and e). Interestingly, HIF-1*α*, well known for its role in tumorigenesis and tumor progression, is a critical regulator in regulation of HK2 and MCT4 during tumor Warburg^27, 28^ and we also found that miR-1 had an effect on HK2, HIF-1*α* and MCT4 mRNA expression levels (Figures 3b, d and f), indicating that these glycolytic metabolic enzymes were regulated by miR-1 may not only at mRNA transcriptional level but also at protein expression level.

### HIF-1*α* is a target of miR-1

**Figure 4:**
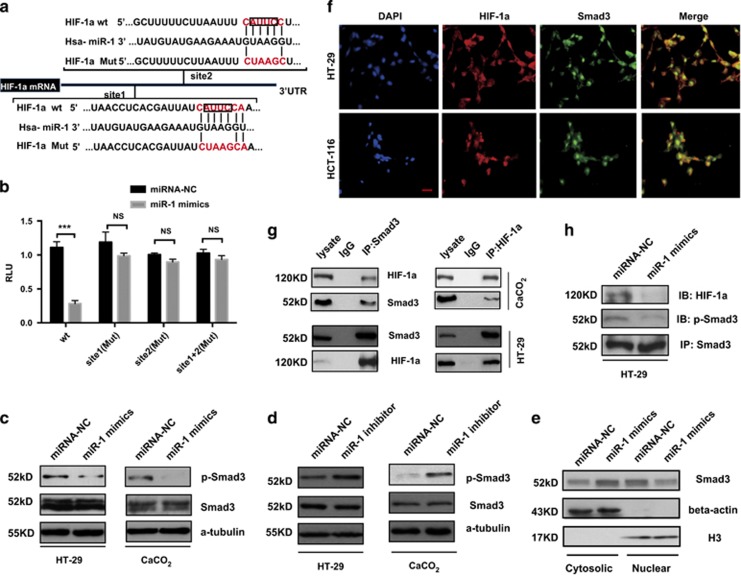
miR-1 silencing contributes to HIF-1*α* expression and phosphorylation of Smad3. (**a**) Predicted base pairing between miR-1 and 3′-UTR of HIF-1*α*. Wild-type or mutated (mut) 3′-UTR of HIF-1*α* sequence inserted into dual-luciferase vector were shown. (**b**) miR-1 mimics were co-transfected with psiCHECK2- HIF-1*α*-3′-UTR or psiCHECK2-HIF-1*α*-3′-UTR-mut, including mut1 and mut2, into HEK293 cells followed by dual-luciferase analysis. Data were presented as mean (±s.d.). *n*=3 in each group. ***P<0.001 as compared with indicated group. (Student’s *t*-test). (**c**,**d**) WB results show that compared with those treated with miR-1 mimics or miR-1 inhibitor transfection leads to changes in the protein levels of p-Smad3 in both HT-29 and CaCO_2_ cells. (**e**) Indicated group of HT-29 cells were extracted to detect nuclear and cytosolic Smad3 by western blotting, beta-actin and H3 were used as internal controls for cytosolic and nuclear fractions, respectively. (**f**) Immunofluorescence of Smad3 and HIF-1*α* localization in HT-29 and HCT-116 cell. (**g**) Smad3 interacted with HIF-1*α*. CaCO_2_ (upper) and HT-29 (bottom) cells were grown to 70–80% confluence, and then whole cell lysates were immunoprecipitated with anti-Smad3 antibodies and coprecipitated with HIF-1*α*. (**h**) The lysis of indicated group were immunoprecipitated with antibody targeting endogenous Smad3 and coprecipitated with HIF-1*α* by immunoblotting

MiR-1 had been associated with the Warburg effect in colorectal tumor cells, but the mechanisms by which miR-1 inhibited glycolysis with affecting HIF-1*α* expression needed exploration. Bioinformatic analysis revealed two potential targets of miR-1 binding in fragments in the 3′-UTR region of the *HIF-1α* gene. A luciferase reporter assay was used to determine whether miR-1 could directly target the 3′-UTR regions of HIF-1*α*. We cloned the target sequences of wild-type (wt) 3′-UTR or mutant (mt) 3′-UTR into a luciferase reporter vector (Figure 4a), and transfected HEK293 cells with the wt or mt 3′-UTR vector and miR-1 mimics. We found that forced expression of miR-1 mimics significantly suppressed the activity of the luciferase reporter gene containing the wt 3′-UTR of HIF-1*α*, but not the gene containing the mutated form (Figure 4b); in contrast, no significant difference in RLU were obtained in psicheck2-MCT4-3′-UTR and psichek2-HK2-3′-UTR compared with control in miR-1 overexpressed HEK293 cells, respectively (Figure 1s). In addition, we transfected siRNA targeted to HIF-1*α* into HT-29 overexpressed miR-1 mimics, which further diminished HK2 and MCT4 expression (Figure 2s). Collectively, these data indicated that HIF-1*α* was a direct target of miR-1 and had a critical role in tumor glycolysis.

### MiR-1 repressed the Warburg effect by inactivation of Smad3

The above results may imply that miR-1 regulated HK2 and MCT4 expression by directly target HIF-1α expression; however, it is still unknown that the miR-1 decreased HIF-1α expression at mRNA level, which further attracted us to uncover the underlying mechanism. Interestingly, previous studies showed that Smad3 is a repressor of PGC-1a expression, and regulates both glucose tolerance and energy homeostasis.^13^ We wondered whether miR-1 has an impact on glycolysis signaling via modulating phosphorylation of Smad3. As shown in Figure 4c, western blots revealed that miR-1 mimics significantly suppressed the Smad3 pathway through decreased phosphorylation of Ser213 on the Smad3 linker region predominant in HT-29 and CaCO_2_ cells, a phosphorylation site central to tumor progression, despite luciferase assay showed that Smad3 is not a target of miR-1 (Figure 1s). while phosphorylation of Smad3 was significantly enhanced in cells transfected with miR-1 inhibitor (Figure 4d). In addition, as shown in Figure 4e, primarily location of Smad3 in nuclear was substantially reduced by miR-1 mimics overexpressed in HT-29 cells, α-tubulin and H3 served as internal control for the cytosolic and nuclear fraction, respectively. Taken together, these results indicated that the critical role of miR-1 in Smad3 nuclear translocation and activity.

### Smad3 regulated and interacted with HIF-1α in turn to promote Smad3 activity

Despite the above results demonstrated that HIF-1α is a target of miR-1, the mechanism through which miR-1 mimics decreased Smad3 activity is still unknown. Next, we focus our attention on the relationship between HIF-1α and Smad3. We found that depletion of Smad3 in colorectal cancer cells reduced HIF-1*α* expression, which was consistent with another work from our team also exhibited that Smad3 contributed to tumor glycolysis, which is key regulator in tumor glycolysis (data not shown, unpublished), these results implied that miR-1 regulated tumor glycolysis not only by directly targeting HIF-1*α* but also by Smad3-mediated HIF-1*α*, HK2 and MCT4 expression.

Importantly, as shown in Figures 4f and g, immunofluorescence and immunoprecipitation showed that endogenous Smad3 interacted and co-localized with HIF-1*α*, which is consistent with previous reports by Rozen-Zvi *et al.*^29^ and Shi *et al.*^30^ the study also demonstrated that the formation of a Smad3/HIF-1*α* transcriptional complex led to increase phosphorylation of Smad3,^30, 31, 32^ which may be a novel interaction in regulation of tumor glycolysis. Most importantly, our results showed that this interaction was dramatically disrupted by transfection of miR-1 mimics (Figure 4h), which may be largely attributed to reduction of HIF-1*α* expression by miR-1 mimics, leading to decrease phosphorylation of Smad3 and inhibit tumor glycolysis. Interestingly, SB431542 treatment as indicated time had no effect on this interaction, but aggravated this inhibition effect of miR-1 mimics (Figure 3s), In summary, these data suggested that Smad3 is the primary regulator of miR-1-induced inhibition of tumor glycolysis.

### MiR-1 had an essential role in the binding of Smad3 to promoters of glycolytic genes

**Figure 5:**
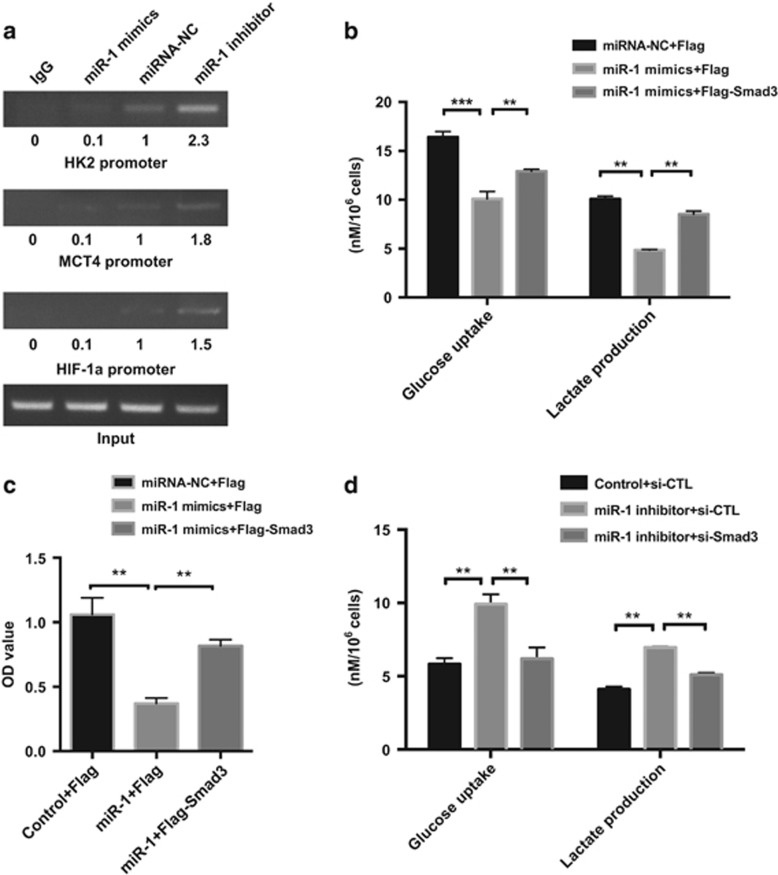
miR-1 is required for Smad3 transactivity and ectopic expression of Smad3 reversed the phenomenon caused by miR-1. (**a**) ChIP analysis of binding of Smad3 protein to indicated genes promoter in HT-29 cells transfected with miR-1 mimics or miR-1 inhibitor. The PCR products were subjected to agarose gel electrophoresis, for each marker band, values were normalized to internal control and the density of control was set at 1.0. (**b**, **d**) glucose uptake and lactate production in supernatant were measured in indicated group in HT-29 cells. Data were presented as mean±s.d., Student’s *t*-test, **P<0.01, ***P<0.001, *n*=3; (**c**) cell proliferation was detected using CCK-8 assay in HT-29 cells after 24-hour transfection as indicated group, Data were expressed as mean±s.d., *n*=5, **P<0.01, Student’s *t*-test

To further confirm the possibility that downregulation of these genes transactivation resulting from miR-1 led to reduction of binding of Smad3 to the promoter sequence, ChIP assays were used to determine whether the binding of Smad3 to HIF-1*α*, HK2 and MCT4 promoters activated transcription in colorectal cancer cells and confirm the functional importance of miR-1 in recruitment of Smad3. A search of target gene promoters on the UCSC Genome Bioinformatics Site found a consensus sequence, CAGACA, which has been identified as a Smad3-binding element (data not shown). PCR assay detected overexpression of promoter fragments in HT-29 cells and pulled down by anti-Smad3 antibody. The promoter fragment containing the Smad3 site was enriched in cells, in which the miR-1 inhibitor was present. Consistent with this, overexpression of miR-1 strongly reduced the binding of Smad3 to the promoter in these cells (Figure 5a). Furthermore, PCR did not detect promoter fragments in IgG group, indicating that the IP and PCR-based amplification of the target gene promoter sequence was specific. Taken together, these results indicated that miR-1 had a significant role in Smad3-mediated transactivation of glycolytic enzymes, including HK2, MCT4 and HIF-1*α*.

### Smad3 overexpression abolished the effect of miR-1 inhibition on glycolysis and cell proliferation

As our results demonstrated that miR-1 regulated tumor glycolysis in colorectal cancer cells via inactivation by Smad3, we investigated whether Smad3 was required for miR-1-mediated inhibition of tumor glycolysis and proliferation. As expected, Smad3 significantly enhanced glucose uptake and lactate production in transfected HT-29 colorectal cancer cells that overexpressed miR-1 mimics (Figure 5b). Cell proliferation analysis revealed that ectopic overexpression of Smad3 diminished the inhibitory effect of miR-1 on cell growth (Figure 5c). Finally, the effects on cell proliferation and glycolysis were not seen in HT-29 and CaCO_2_ cells expressing miR-1 inhibitor and Smad3 downregulation (Figure 5d). These results demonstrated that Smad3 is required for miR-1-mediated suppression of tumor proliferation and glycolysis in colorectal cancer cells.

### Functional role of the miR-1/Smad3/HIF-1*α* axis in regulating glucose metabolism and controlling tumorigenesis *in vivo*

**Figure 6:**
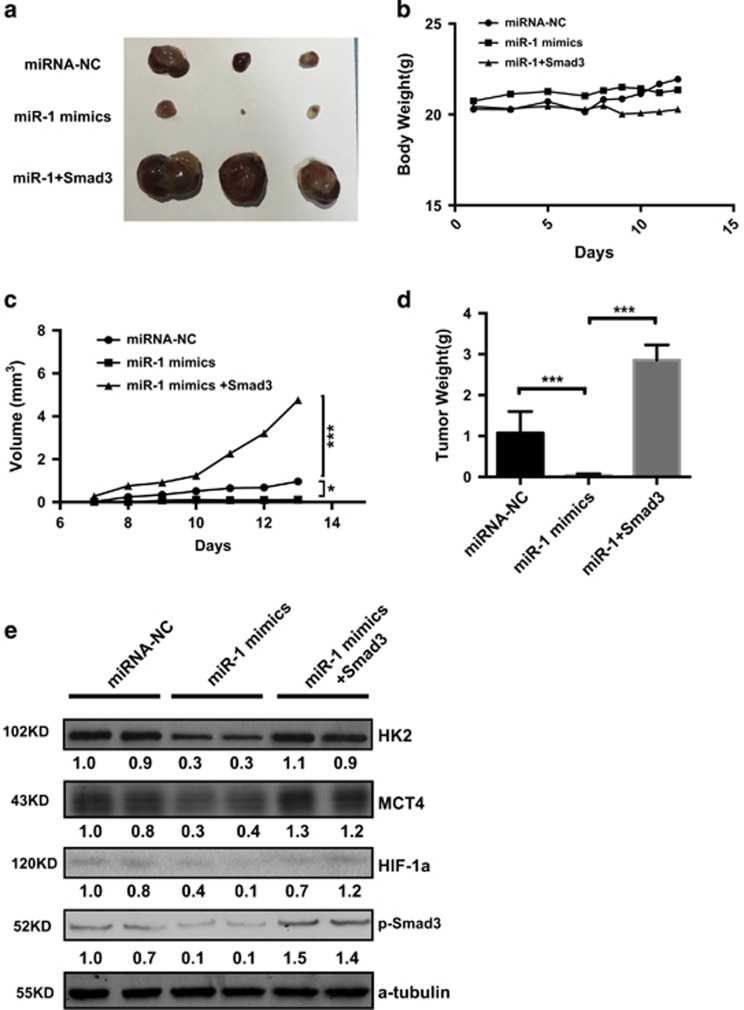
Functional role of the miR-1/Smad3 axis in regulating tumor glycolysis and proliferation *in vivo*. *In vivo* null mice xenograft study. The null mice carrying tumors and tumor sizes was measured for 2 weeks, excised tumor images (**a**), body weight (**b**) and tumor volume (**c**) and tumor weight (**d**) from respective groups are represented (*n*=3). Data were presented as mean±s.d. *P<0.05; **P<0.01 and ***P<0.001, Student’s *t*-test (**e**) Protein expression in tumor from indicated group was determined by western blotting, for each marker band, values were normalized to internal control and the density of control was set at 1.0

Given the importance of miR-1 in aerobic glycolysis in cancer cells, the results prompted us to examine whether activity of the miR-1/Smad3/ HIF-1*α* axis influenced tumor growth *in vivo*. HT-29 cells were injected into nude mice as indicated group, and body weight, tumor growth and tumor volume was monitored. As shown in Figures 6a and d, cells expressing the miR-1 mimic generated smaller tumors than control group and those overexpressing Smad3, indicating miR-1 suppressed, while Smad3 promote tumor proliferation *in vivo*. Importantly, western blots revealed that, compared with control group, HK2, MCT4, HIF-1*α* and pSmad3 protein expression was significantly reduced in cells with miR-1 mimics. Downregulation of HK2, MCT4 and HIF-1*α* was partially reversed when Smad3 was induced (Figure 6e). Taken together, these results are consistent with regulation of cell proliferation and glycolysis in colorectal cancer cells by a cascade controlled by the miR-1/Smad3/HIF-1*α* axis.

## Discussion

**Figure 7:**
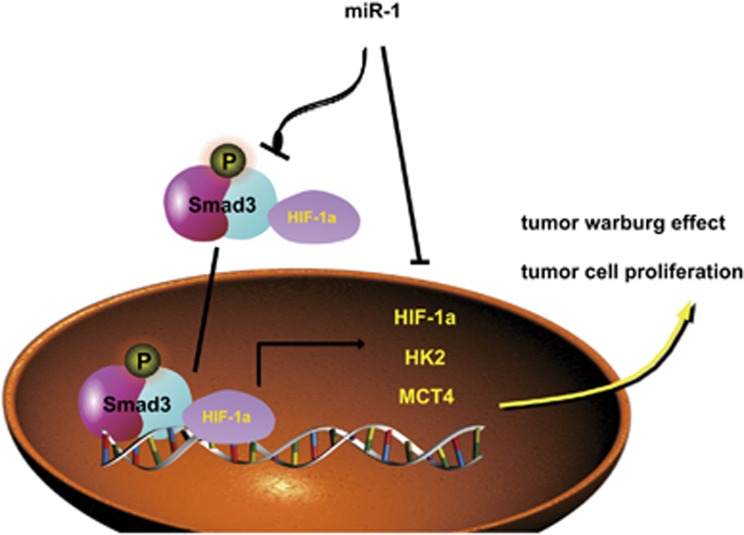
A model diagram for regulation of proliferation by miR-1–Smad3 axis-mediated tumor glycolysis in colorectal cancer. On one hand, miR-1 mediates HIF-1*α* depletion by targets 3′-UTR of HIF-1*α*, which further attenuates tumor glycolysis and inhibits proliferation. On other hand, miR-1 significantly abolishes the interaction between Smad3 and HIF-1*α*, which are attributed to reduction of HIF-1*α*, leading to suppress activation of Smad3 and reduce the expression of metabolic enzymes in the Warburg effect, such as HIF-1*α*, HK2 and MCT4, finally inhibits tumor proliferation. Interestingly, Smad3 interacts and regulates HIF-1*α* expression, indicating a critical positively feedback loop in tumor progression

Colorectal cancer is the most common malignant neoplasm worldwide. Despite efforts to improve cancer management, increasing overall survival in advanced and metastatic disease is still a challenge.^33^ The molecular mechanisms underlying the development of colorectal cancer are not well understood, and a better understanding of tumor formation and progression will help to discover novel therapeutic targets and develop novel strategies for the treatment of human colorectal cancers. As shown in Figure 7, we demonstrated that miR-1 suppressed aerobic glycolysis while Smad3 promoted it via its downstream effecter HIF-1*α* and glycolytic enzymes, including HK2, MCT4, in colorectal cancer. We also demonstrated that suppression of tumor glycolysis by the miR-1/Smad3 axis was critical for inhibition of tumor cell proliferation *in vivo*. We thus report a previously unappreciated mechanism to explain, at least partially, the function of miR-1 in inhibition of tumor cell proliferation. These findings greatly extend our insight into the molecular functions of the miR-1/Smad3 axis in tumor progression.

MiRNAs are involved in the pathogenesis of colorectal cancer and negatively regulate gene and protein expression by acting as oncogenes or tumor suppressors. Multiple studies have found that miR-1 is downregulated in many cancers compared with the corresponding normal tissue, including lung and head and neck sarcomas in addition to colorectal cancer.^34, 35, 36, 37^ For example, our finding of miR-1 inhibition of colorectal cancer cell growth is similar to results reported by Kojima *et al.*^38^ and Han *et al.*^39^ that ectopic overexpression of miR-1 suppressed cell proliferation, migration, wound healing, and invasion of gastric cancer cells. Ectopic miR-1 expression has been shown as a candidate tumor suppressor in other human cancers.^40^ The antiproliferation activity of miR-1 and its effect on tumor progression have been previously reported. However, the role of miR-1 in tumor glycolysis and the molecular mechanism through which miR-1 regulates the Warburg effect to affect cell proliferation were not known. We showed that miR-1 inhibited tumor proliferation by suppression of glycolysis and negative regulation of Smad3 activity and HIF-1*α* expression.

Smad3, a critical downstream component of TGF-*β* signaling, consists of two highly conserved MAD homology domains (MH1 and MH2) connected by a less conserved linker domain.^41^ Phosphorylation of the Smad3 linker domain, which contains threonine and serine residues Thr179, Ser204, Ser208 and Ser213, is mediated by MAPK, CDK and GSK3. Phosphorylation, particularly at Ser213,^12, 42, 43, 44^ significantly blocks TGF-*β* inhibitory effect. Phosphorylation of Ser213 has been shown to transmit malignant TGF-*β* signals in  advanced stages of human colorectal cancer,^6^ thereby altering tumor-suppressive TGF-*β* signaling in those malignant cells. Linker phosphorylation of Smad2 and Smad3 may represent a target for intervention in human metastatic cancer. In this study, we investigate the actions of phospho-Smad3 Ser213 on miR-1 mediation of tumor glycolysis. We found that phospho-Smad3 Ser213 was decreased in HT-29 and CaCO_2_ cells expressing miR-1, which further inhibited Smad3 binding to the promoter thereby suppressing translation. Overexpression of Smad3 significantly reversed the inhibition of miR-1-mediated tumor glycolysis and tumor growth. These finding indicated Smad3 as a critical regulator of tumor progression.

In the hypoxic tumor microenvironment, HIF-1*α* protein escapes degradation and translocates into the nucleus, where it initiates a gene expression program that leads to a switch from oxidative phosphorylation to glycolysis. HIF-1*α* target genes include glucose transporters that increase glucose uptake and pyruvate dehydrogenase kinases (PDK1–3) that shunt pyruvate away from mitochondria through the inhibition of pyruvate dehydrogenase.^45, 46, 47, 48, 49^ In this study, we found that HIF-1*α* was a direct functional target of miR-1 in human colorectal cancer. Downregulation of miR-1 significantly increased HIF-1*α* expression, resulting in enhancement of tumor glycolysis and tumor growth. In addition to PKM2 and STAT3, we also found that Smad3 interacted with HIF-1*α* and increased activity of Smad3. Ectopic expression of miR-1 mimics markedly abolished this interaction, which largely be attributed to inhibit HIF-1α expression caused by miR-1, resulting in reduction of phosphorylation of Smad3. Based upon these finding, HIF-1*α* may be proposed to be part of a ‘feed forward loop’ enhancing the activity of Smad3, a key transcriptional regulator of both aerobic and anaerobic glycolysis, further research is needed to determine this speculation in our work.

In summary, our study is the first to unravel the role and mechanism of miR-1 in tumor cell proliferation by regulation of tumor glycolysis in colorectal cancer. MiR-1 suppressed glucose uptake, lactate production and cell proliferation by inhibition of HIF-1*α* expression, which further uncoupling from Smad3 and dephosphorylation of Ser213 in the Smad3 linker domain, finally the subsequent reduction of Smad3 binding to the promoter and decreased HIF-1*α* transactivity, and attenuated cell proliferation. We identified HIF-1*α* is a direct target of miR-1, and most important, Smad3 regulated and interacted with HIF-1*α*, leading to increasing Smad3 activity, which implies the contribution of a feed forward loop in regulation of tumor glycolysis and proliferation. Further studies are required to elucidate the relationship between Smad3 and HIF-1*α*, such as the effect of this interaction on HIF-1*α* stabilization, during tumor glycolysis and tumor progression. However, the current study provides novel insights into the functional role of miR-1 in tumor glycolysis.

## Materials and Methods

### Chemicals and reagents

Reagents were obtained from Sigma (St. Louis, MO, USA), Lipofectamine 3000 for siRNA transfection was from Invitrogen (Thermo Fisher Scientific, Carlsbad, CA, USA), and HilyMax for plasmid transfection was from Dojindo (Kamimashikigun, Kumamoto, Japan). All-in-One First-Strand cDNA Synthesis Kits and All-in-One qPCR Mix were from GeneCopoeia (Rockville, MD, USA), and miRNA real-time PCR (RT-PCR) kits were from Genepharma (Shanghai, China). Luciferase assay kits were from Promega (Madison, WI, USA) and ChIP-IT Express Enzymatic Chromatin Immunoprecipitation Kits were from Active Motif (Carlsbad, CA, USA). SB431542 was purchased from Calbiochem/EMD (La Jolla, CA, USA). Antibodies used for immunoblotting and immunoprecipitation (IP) assays from Cell Signaling Technology (Danvers, MA, USA), Smad3 antibody for chromatin IP and western blotting from Santa Cruz Biotechnology (Santa Cruz, CA, USA), and Alexa 488- and 594-conjugated secondary antibodies from Molecular Probes (Invitrogen, Thermo Fisher Scientific). Antibodies targeting phosphorylated Smad3 at Ser213, HIF-1*α* and all unconjugated secondary antibodies were from Abclonal (Wuhan, China).

### Cell lines and cell culture

HCT-116, SW480, SW620, HT-29 human colon adenocarcinoma cells, CaCO_2_ cells and HEK293 human embryonic kidney cells were maintained in Dulbecco's Modified Eagle Medium (DMEM) supplemented with 10% fetal bovine serum (Invitrogen-GIBCO), while NCM460 were cultured in RPMI1640. All cells were cultured in a humidified incubator at 37 °C and 5% CO_2_.

### Cell transfection

For *in vitro* transfection, cells were seeded into 24-well plate at a density of 3.0 × 10^5^ cells per ml in 500 *μ*l DMEM medium supplemented with 10% FBS. After incubation for 24 h, miRNA mimics and recombinant plasmids were transfected transiently into cells using Lipofectamine3000 and Hilymax, receptively, following the kit manufacturers’ instructions, the following siRNA sequences were used: HIF-1*α*, 5′–3′-CUGAUGACCAGCAACUUGA.

### Generation of stable cell line

The lentivirus vectors Lv-miRNA-1 mimics, LV-miR-1 inhibitor and LV-miRNA-NC were purchased from Genepharma. Puromycin was purchased from Sigma and used to select for stably transfected cells.

### Lactate and glucose assays

Glucose and lactate concentrations were measured in subconfluent cell culture growing in 12-well plate. Lactate and glucose concentration in the culture media was measured for 24 h using Lactate assay kit from sigma (St. Louis, MO, USA) and Nova Biomedical BioProfile 100 plus machine, respectively, then finally normalized against the number of cells in each well.

### Cell proliferation assay

Cell proliferation was measured with a Cell Counting Kit-8 (CCK-8) (Dojindo). Briefly, cells were seeded into 96-well plate at a concentration of 5 × 10^3^ cells per well. Each well contained 10 *μ*l CCK-8 in 90 *μ*l of culture medium. The cells were incubated for 1 h at 37 °C, and absorbance were measured at 450 nm. The assays were performed in triplicate.

### RNA isolation and real-time quantitative PCR

Total RNA was isolated from cultured cells using TRIZOL reagent (Invitrogen), and cDNA was synthesized using an All-in-One First-Strand cDNA Synthesis Kit. The relative expression of HIF-1*α*, HK2 and MCT4 was detected using an All-in-One qPCR Mix. Real-time PCR assay of miR-1 expression was performed on an Applied Biosystems StepOnePlus system, using a SYBR Green I Real-Time PCR kit (GenePharma). Sequences of the primers used are shown in Supplementary Table S1.

### Bioinformatic predictions

RNAhybrid and TargetScan7.1 are online software programs that were used for miRNA target prediction.

### Luciferase assays

Cells were co-transfected with the plasmid psicheck2 dual-luciferase reporter vector containing 3′-UTR or 3′-UTR-mut of HIF-1*α*, HK2, MCT4 or Smad3 along with miR-1 mimics or negative controls. The cells were washed with phosphate buffered saline and lysed after 48 h in passive lysis buffer (1 × PLB). The relative light units of stimulated cells were measured using the Luciferase Reporter System (Promega). Renilla luciferase was normalized to firefly luciferase activity.

### Co-immunoprecipitation (Co-IP)

When 80% confluent, HT-29 or CaCO_2_ cells were lysed in NP-40 lysis buffer and precleared with protein G-sepharose beads for 15 min. Lysates were then incubated with anti-Smad3, HIF-1*α*, or nonspecific immunoglobulin (IgG), in the presence of protein G-sepharose beads overnight at 4 °C. The beads were washed with ice cold NP-40 buffer. Elution was performed by adding 2 × SDS-PAGE protein sample buffer and boiling at 95 °C for 10 min. Protein expression was assayed in western blots.

### Subcellular fractionation and western blot assay

Subcellular fractionation was conducted using the Membrane and Cytosol Protein Extraction Kit from Beyotime following the manufacturer’s recommendations. Total protein was extracted from 2 × loading buffer, and protein concentration was measured with the Bio-Rad assay system (Bio-Rad, Hercules, CA, USA). Equal amounts of protein were separated by SDS-PAGE. Antibodies against Smad3, pSmad3 (Ser213), MCT4, HK2 and HIF-1*α* assayed by blotting, *α*-tubulin used as an internal control.

### Immunofluorescence microscopy

Cells were seeded on cover slips in a six-well plate and allowed to attach overnight. Immunofluorescent staining was performed as previously described. Fixed and permeabilized cells were incubated overnight at 4 °C with primary antibodies, followed by incubation with Alexa-488- or Alexa 594-conjugated secondary antibodies for 1 h at room temperature. The cover slips were then mounted onto glass slides with prolong gold antifade reagent after staining the nuclei with diamidinophenylindole, the cells were imaged by laser scanning confocal fluorescent microscopy.

### Chromatin immunoprecipitation (ChIP)

ChIP was carried out using an anti-Smad3 antibody (Santa Cruz Biotechnology) or mouse IgG (Millipore, Billerica, MA, USA) and chromatin extracts equivalent to 2 × 10 cells. ChIP samples were quantified by qPCR (SYBR Green Master Mix: Applied Biosystems) and the ChIP qPCR data were normalized using the percent input method. The primer were listed in Supplementary Table S1.

### Mice

All animal experiments were approved by the Guangzhou Women and Children’ Medical Center and Southern Medical University animal care and use committee.

### Statistical analysis

All analysis was conducted using GraphPad Prism V software (La Jolla, CA, USA). A *P-*value <0.05 was considered statistically significant. Statistical differences among groups were determined by Student’s *t*-test, one sample *t*-tests were used to determine the significance of between-group differences in RT-PCR results.

## Supplementary Material

Supplementary Figure 1S

Supplementary Figure 2S

Supplementary Figure 3S

Supplementary Figure legend

Supplementary Table 1S
