# Supplementary figures and images for: MiR-1 suppresses tumor cell proliferation in colorectal cancer by inhibition of Smad3-mediated tumor glycolysis
Source: Cell Death Dis. 2017 May 4;8(5):e2761–. doi: 10.1038/cddis.2017.60 (PMC5520746; doi:10.1038/cddis.2017.60)

**Fig.1s**

**A**

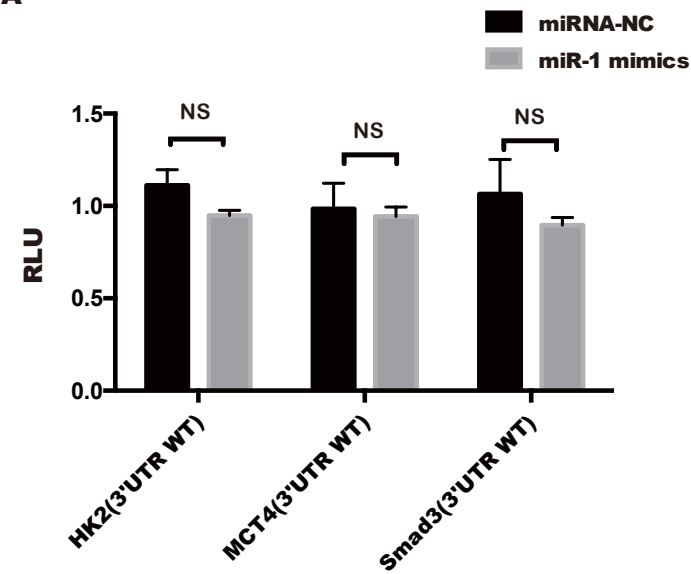

Supplement: Supplementary Figure 1S [file cddis201760x1.pdf]

**Fig.2s**

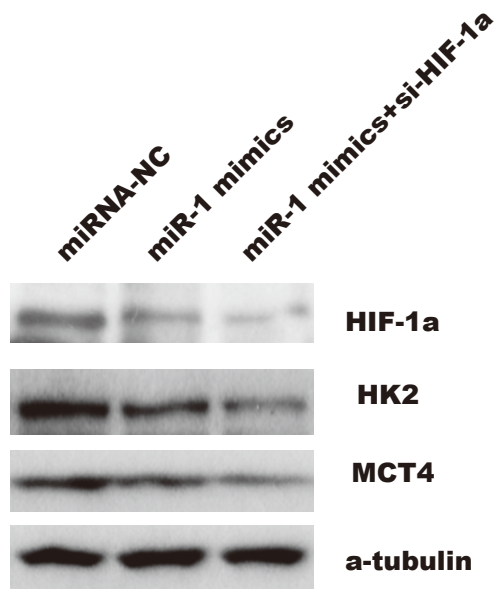

Supplement: Supplementary Figure 2S [file cddis201760x2.pdf]

**Fig.3s**

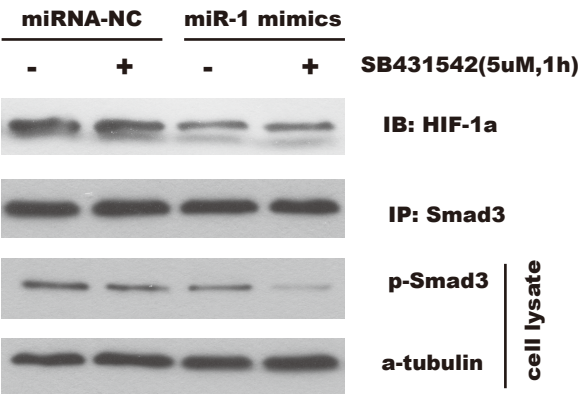

Supplement: Supplementary Figure 3S [file cddis201760x3.pdf]
