# Supplementary material for: MiR-1 suppresses tumor cell proliferation in colorectal cancer by inhibition of Smad3-mediated tumor glycolysis
Source: Cell Death Dis. 2017 May 4;8(5):e2761–. doi: 10.1038/cddis.2017.60 (PMC5520746; doi:10.1038/cddis.2017.60)
Supplement: Supplementary Figure legend [file cddis201760x4.pdf]

Supplementary Figure legend

Fig.1s Indicated genes were not targets of miR-1. The 3'UTR of indicated genes were cloned into psicheck2 plasmid and sequenced by IGene company (China,Guangzhou), which were co-transfected into HEK293 cells with miR-NC and miR-1 mimics, respectively. 48 hours after transfection, the relative luciferase unit were performed as instruction.

Fig.2s depletion of HIF-1a expression aggravated the miR-1 inhibition effect. siRNA targeted HIF-1a were transfected into indicated stable HT-29 cell, 60 hours after transfection, the whole cell lysis were harvested and performed to detect indicated proteins by western blotting.

Fig.3s SB431542 treatment decreased phosphorylation of Smad3 rather than dissociation Smad3 from HIF-1a. Indicated group from HT-29 were grown to 70–80% confluence and serum starved for 24 hours, then stimulated for another 1 hour, the whole cell lysates were immunoprecipitated with anti-Smad3 antibodies, western blotting were performed to analyze the change of HIF-1a expression.
