# Supplementary material for: MiR-1 suppresses tumor cell proliferation in colorectal cancer by inhibition of Smad3-mediated tumor glycolysis
Source: Cell Death Dis. 2017 May 4;8(5):e2761–. doi: 10.1038/cddis.2017.60 (PMC5520746; doi:10.1038/cddis.2017.60)
Supplement: Supplementary Table 1S [file cddis201760x5.pdf]

Supplementary Materials

Table S1

1. Real-time primer:

Genes	QPCR Forward primer	QPCR Reverse primer
HK2	5’-ACCTTTGTGAGGTCCACTCC-3’	5’-TGTCCGTTACTTTCACCCAA-3’
SLC16A4	5’-GGGCTTTCGTTGTTACTGGT-3’	5’-GGTAGTTACCACAGCAGCCA-3’
HIF-1α	5’-AAGGTATTGCACTGCACAGG-3’	5’-AATGGGTTCACAAATCAGCA-3’

2. ChIP primer:

Genes	ChIP Forward primer	ChIP Reverse primer
HK2	5’-CATGCGTCAGGCACCATTTT-3’	5’-GTCTGGTCCCGAGTCTTAGC-3’
SLC16A4	5’-GGGGAGTGTGGGCCATTATT-3’	5’-CACCATCGTCTGTCCTCACC-3’
HIF-1α	5’-GGTCACTTCCTCCCACCTAAT-3’	5’-CAGGCTCACGCTACGGAATC-3’
